# Purpura Fulminans Due to Escherichia coli Septicemia in Relapsed Acute Myeloid Leukemia: An Unusual Case of Protein C Deficiency and Disseminated Intravascular Coagulation

**DOI:** 10.7759/cureus.97951

**Published:** 2025-11-27

**Authors:** Tarek Zieneldien, Sophia Ma, Karanbir Singh, Adeshpal Singh, Janice Kim, Ana Velez, John Greene

**Affiliations:** 1 School of Medicine, Johns Hopkins University, Baltimore, USA; 2 Internal Medicine, Government Medical College, Amritsar, IND; 3 College of Osteopathic Medicine, Michigan State University, East Lansing, USA; 4 Infectious Disease, Moffitt Cancer Center, Tampa, USA; 5 Internal Medicine, Moffitt Cancer Center, Tampa, USA

**Keywords:** acute myeloid leukemia, critical care dermatology, disseminated intravascular coagulation, escherichia coli, immunocompromised host, protein c deficiency, purpura fulminans, sepsis

## Abstract

Purpura fulminans (PF) is a rare and life-threatening condition characterized by rapid development of cutaneous microvascular thrombosis and skin necrosis, typically associated with disseminated intravascular coagulation (DIC). Here, we present a case of PF in an adult patient with relapsed acute myeloid leukemia (AML) who developed *Escherichia coli (E. coli)* septicemia. The patient’s coagulopathy, including marked protein C deficiency, and immunosuppression required resuscitation, targeted antibiotic therapy, correction of hemostatic abnormalities, and surgical management. This case highlights the challenges of managing protein C deficiency and immunosuppression in PF, demonstrating how these interventions are critical to preventing progression and improving outcomes in this complex context.

## Introduction

Purpura fulminans (PF) is a rare, life-threatening thrombotic disorder characterized by cutaneous hemorrhagic necrosis, progressive acral ischemia, and vascular thrombosis in the setting of systemic coagulopathy [1]. It is most often seen as a complication of disseminated intravascular coagulation (DIC) triggered by overwhelming sepsis, with *Neisseria meningitidis *and* Streptococcus pneumoniae *being the most common organisms [1]. PF can also occur in association with an inherited or acquired protein C deficiency, trauma, or malignancy [2]. The pathophysiology involves a consumptive coagulopathy driven by the depletion of natural anticoagulant proteins, particularly protein C, and its cofactor, protein S, which normally function to downregulate thrombin generation and prevent microvascular thrombosis. The dermatologic manifestations, such as purpuric plaques, hemorrhagic bullae, and gangrene, serve as cutaneous markers of systemic DIC [3].

In immunocompromised patients, the risk of DIC and PF is increased due to impaired host defense mechanisms, prolonged neutropenia, and a predisposition to invasive infections [4]. Acute myeloid leukemia (AML), in particular, carries a high risk of septic complications [5]. Both the disease itself and the treatment with intensive chemotherapy regimens, such as cytarabine and anthracyclines, lead to increased immunosuppression, disruption of mucosal barriers, and colonization with multidrug-resistant organisms [5].

Here, we present the case of a patient with relapsed AML who developed septic shock secondary to *E. coli *bacteremia complicated by DIC and PF. In *E. coli* sepsis, lipopolysaccharide endotoxin activates monocytes and endothelial cells, triggering the release of proinflammatory cytokines (TNF-α, IL-1β) that upregulate tissue factor expression and accelerate coagulation. The patient's case was notable for viral coinfections, acral gangrene, and compartment syndrome, highlighting PF as a severe but underrecognized manifestation of gram-negative sepsis in immunocompromised patients. PF secondary to *E. coli* sepsis remains underrecognized, especially in adult hematologic malignancies, where diagnostic delay can lead to catastrophic outcomes. While PF is a recognized but rare syndrome characterized by consumptive coagulopathy, its occurrence in the setting of relapsed AML complicated by gram-negative septicemia is exceptionally uncommon. This case highlights the diagnostic and management challenges in this unique context and provides insights into clinical presentation and laboratory findings, contributing to the existing literature. Our objective is to describe the clinical course, diagnostic challenges, and multidisciplinary management of PF in the setting of gram-negative sepsis and hematologic malignancy.

## Case presentation

**Figure 1:**
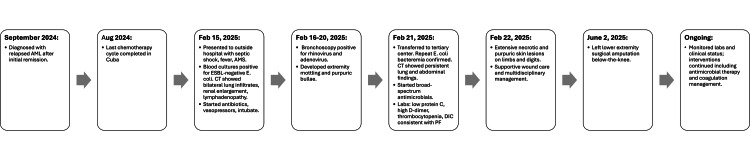
Timeline of patient prognosis and management.

A 25-year-old Hispanic female with a history of AML, diagnosed with relapsed AML in September 2024, presented to our cancer centre in February 2025 with septic shock, altered mental status, and respiratory failure (Figure 1). She had previously undergone induction and consolidation chemotherapy with cytarabine and daunorubicin, with her last treatment administered in Cuba in August 2024. Upon presentation, the patient exhibited laboratory findings consistent with relapsed AML, including severe anemia (hemoglobin 7.2-7.4 g/dL), profound thrombocytopenia (platelets as low as 6,000/μL), and an abnormal white blood cell count (ranging from 1.6 to 13.03 K/mm³). The diagnosis of AML and relapse were corroborated by hematology-oncology notes, cytogenetic analysis demonstrating trisomy 8, a pathogenic ASXL1 mutation, and bone marrow findings (5/16 cellularity). These hematologic abnormalities, combined with her history of intensive chemotherapy, resulted in immunosuppression that predisposed her to invasive *E. coli* septicemia and disseminated intravascular coagulation, ultimately leading to PF. The patient had achieved remission but relapsed in September 2024. 

**Figure 2:**
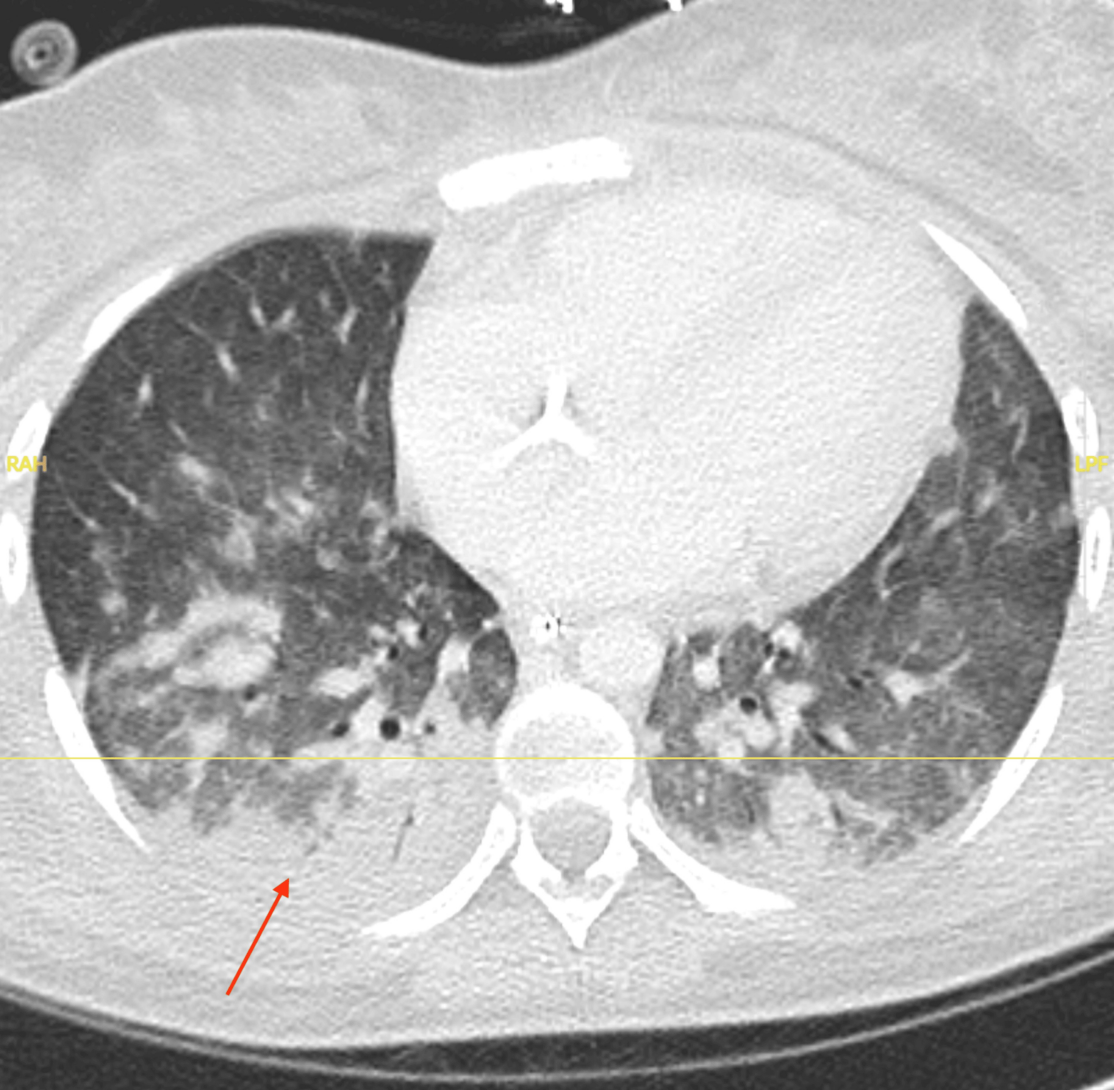
CT scan image of the thorax. The figure shows bilateral consolidation infiltrates in the lung bases. Innumerable ill-defined nodular ground-glass opacities are seen bilaterally in the lungs, likely representing atypical infiltrates. There is also underlying pulmonary venous congestion and mild interstitial edema. Extensive bibasal atelectasis is noted. Axial CT images are displayed in the standard anatomical orientation, with the patient's anterior (front) positioned toward the top of the image and the posterior (back) toward the bottom. CT was obtained on 02/21/2025.

She presented to an outside hospital on February 15, 2025, with complaints of altered mental status, body aches, fever (100.3°F), and low blood pressure (102/47mmHg). Blood cultures obtained there revealed ESBL-negative *E. coli *bacteremia. She tested negative for HIV, Hepatitis B, Hepatitis C, and Adenovirus. A CT scan of the thorax demonstrated bilateral consolidation infiltrates in the lung bases (Figure 2). Additional findings included bilateral renal enlargement, bulky right external iliac adenopathy (largest 3.5 × 2.8 cm), and pelvic/retroperitoneal lymphadenopathy. A diagnosis of septic shock due to *E. coli* bacteremia with acute kidney injury and pneumonia was made. She received aggressive fluid resuscitation, vancomycin, cefepime, and vasopressors, but continued to deteriorate, requiring intubation and transfer to the critical care unit.

During her admission, she underwent bronchoscopy for clearance of thick secretions; the respiratory viral panel was positive for rhinovirus and adenovirus. She subsequently developed bilateral upper and lower extremity mottling with purpuric bullae, for which nitroglycerin patches were applied but without any relief. Due to progressive clinical decline, she was transferred to our hospital on February 21, 2025, for higher-level care.

Upon arrival, the patient was afebrile, tachycardic, hypotensive, and was intubated. All pertinent labs, including blood and urine cultures, were repeated, and again confirmed *E. coli *ESBL-negative bacteremia. Laboratory investigations from both facilities are summarized in Table 1.

**Table 1:** Comparative laboratory findings: referring hospital vs. transfer hospital ∗Indicates lab value either not drawn or not available due to error.

Category	Parameters	Laboratory values at presentation to the outside hospital	Laboratory values at presentation to our hospital	Units (normal range)
Hematology	White blood cells	1.6	13.03	3.4-10.8 K/mm³
	Red blood cells	2.35	2.31	4.2–5.9 million/μL
	Hemoglobin	7.4	7.2	Female: 12–16 g/dL; male: 14–18 g/dL
	Hematocrit	22.4	21.2	35.5-44.9%
	Mean corpuscular volume	∗	91.8	80–98 fL
	Mean corpuscular hemoglobin	∗	31.2	28–32 pg
	Mean corpuscular hemoglobin concentration	If∗	34.0	33–36 g/dL
	Red blood cell distribution width	∗	53.7	9.0%–14.5%
	Platelets	6000	53000	150,000–450,000/μL
Coagulation profile	Prothrombin time	26.9	20.5	11-13.5 seconds
	International normalized ratio	2.5	1.8	0.8-1.2
	Activated partial thromboplastin time	∗	29.7	25-35 seconds
	Fibrinogen	∗	262	200-400 mg/dL
	D-dimer	∗	66343	<500 ng/mL
	Thrombin-anti-thrombin complex(TAT)	∗	35.61	<4 ng/mL
	Protein C	∗	37	70-130%
	Protein S	∗	77	60-123%
	Factor V activity	∗	62%	60%–130%
	Factor VII	∗	32	60%–130%
	Factor VIII	∗	447	50%–150%
	Factor X activity	∗	83%	60%–130%
Renal function tests and electrolytes	Blood urea nitrogen	53	15	5-20 mg/dL
	Creatinine	3.10	0.9	0.61.2 mg/dL
	Sodium	127	136	135-145 mmol/L
	Potassium	∗	3.9	3.5-5 mmol/L
	Chloride	∗	106	95-105 mmol/L
	Calcium	6.3	7.8	8.6–10.2 mg/dL
	Magnesium	∗	1.9	1.6–2.6 mg/dL
Liver function tests	Aspartate aminotransferase	298	647	0-40 U/L
	Alanine transferase	90	912	0-32 U/L
	Alkaline phosphatase	67	127	30–120 U/L
	Total bilirubin	∗	12.1	0.0-1.2 mg/dL
	Total protein	∗	4.0	5.5–9.0 g/dL
	Albumin	∗	2.2	3.5-4.8 g/dL
Other markers	Creatine kinase	∗	5834	22-198 U/L
	Lactate dehydrogenase	∗	1617	80–225 U/L
	Lactic acid	6.1	3.8	0.7–2.1 mmol/L
	Procalcitonin	∗	25.79	≤0.10 ng/mL
	Haptoglobin	∗	34	41-165 mg/dL

Repeat CT imaging showed similar findings with an additional dilated sigmoid colon and cecum. She was started on cefepime, metronidazole, linezolid, and micafungin. Although Protein C antigen concentration, fibrin degradation products (FDP), and fibrinogen levels were not measured, the diagnosis of PF was supported by markedly reduced protein C activity (37%), severely elevated D-dimer levels, thrombocytopenia, and coagulation abnormalities consistent with DIC (Table 1). Given the urgency of the patient's clinical condition, management was guided by the available coagulation and clinical data. 

**Figure 3:**
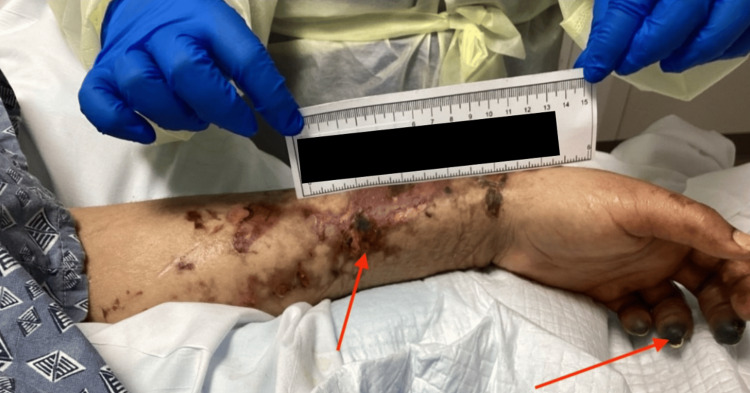
Left forearm and hand with necrotic and purpuric changes.

**Figure 4:**
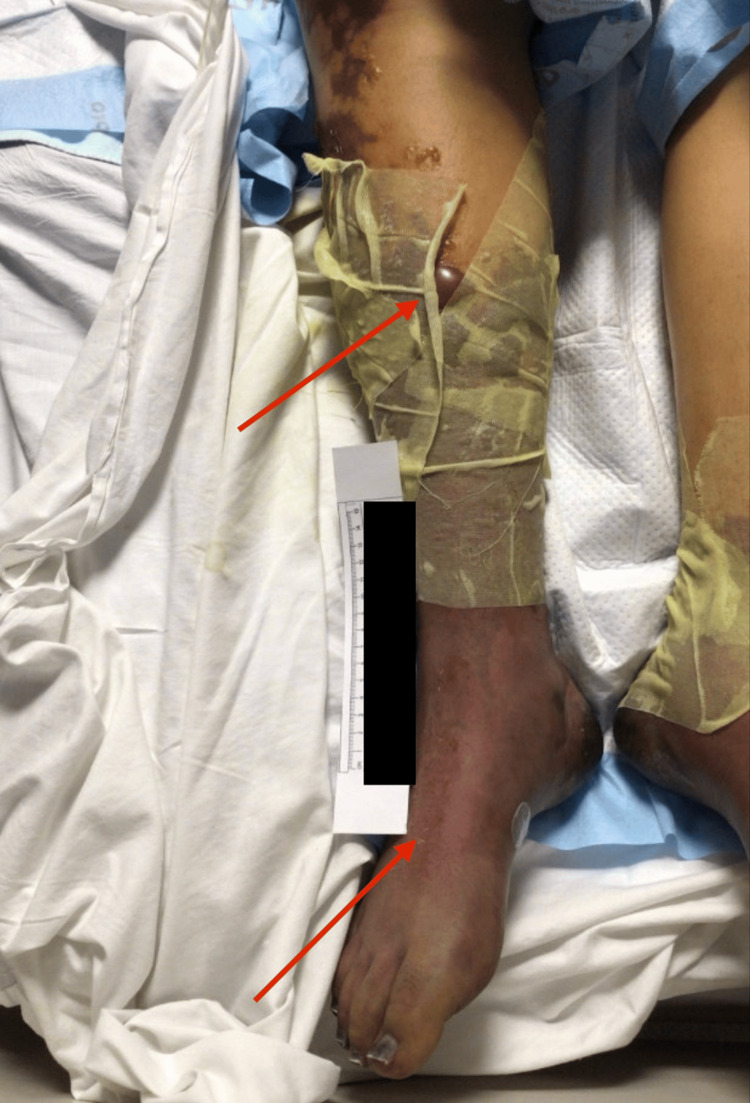
Right leg with purpuric changes and bullae. Dressings included Silverlon, negative-pressure wound therapy (wound VAC) monitored by ViOptix. Betadine was applied to the digits, Silvadene was applied to the contralateral leg, and Webril/ACE compression; a Jackson-Pratt (JP) drain was also placed.

Physical assessment at our facility revealed extensive cutaneous findings: mottled skin, diffuse petechiae on all extremities and trunk, blisters on upper and lower limbs, and necrosis involving the fingers and toes (Figures 3, 4). Peripheral pulses were absent in the distal extremities. There was generalized anasarca with abdominal distension. Notably, the skin remained warm and dry, while the distal digits were cold and necrotic.

The constellation of anemia, thrombocytopenia, abnormal coagulation parameters, markedly elevated D-dimer, and reduced protein C levels in the context of *E. coli *sepsis and DIC (as shown in Table 1) led to the diagnosis of PF. A temporal correlation between laboratory abnormalities and cutaneous necrosis was observed in this patient. Markedly elevated D-dimer levels and severely reduced protein C activity coincided with the onset and progression of purpuric skin lesions, reflecting the evolving DIC underlying PF. While vasopressor use posed a potential confounding factor, the warm appearance of necrotic skin and absent distal pulses supported microvascular thrombosis rather than ischemic necrosis from vasopressor-induced vasoconstriction. Management decisions, including escalation of anticoagulant support and broad-spectrum antimicrobial therapy, were guided by these dynamic laboratory and clinical changes. Comparatively, this case aligns with previously reported *E. coli*-associated purpura fulminans, which similarly demonstrate rapid coagulopathy progression and high morbidity, underscoring the unique challenges in immunocompromised hosts.

## Discussion

PF represents an acute purpuric rash characterized by microvascular thrombosis, hemorrhagic necrosis, and DIC. While classically linked to meningococcemia or pneumococcal sepsis, PF can arise in a variety of infectious and non-infectious contexts, including gram-negative bacteremia, malignancy, and acquired protein C pathway deficiencies. Early recognition of PF is critical, as delayed treatment worsens morbidity and mortality [6]. Management requires a multidisciplinary approach involving critical care, infectious disease, vascular surgery, hematology, reconstructive surgery, and rehabilitation or wound care teams, particularly for tissue debridement or, in severe cases, limb salvage or amputation [1]. Despite aggressive care, PF carries a high mortality rate, often exceeding 40%, with survivors frequently suffering long-term sequelae such as amputation, chronic pain, and functional disability [7].

To our knowledge, fewer than 15 adult cases of PF caused by *E. coli* have been reported, the majority occurring in patients with underlying hematologic or solid malignancies. Outcomes are generally poor, with mortality rates exceeding 50%. This case highlights several clinically important features. PF is typically described in pediatric populations or in post-infectious settings such as meningococcemia, making its occurrence in an adult with gram-negative sepsis and hematologic malignancy an uncommon and noteworthy presentation. While *E. coli *bacteremia is a well-recognized cause of septic shock, it remains a relatively uncommon precipitant of PF, though previously reported in case reports [8-11].

In this patient, the septicemia occurred in the setting of relapsed AML undergoing cytotoxic chemotherapy, resulting in profound immunosuppression characterized by neutropenia and mucosal barrier breakdown, predisposing her to invasive gram-negative infections. In PF associated with relapsed AML and septicemia, the coagulopathy arises primarily from acquired mechanisms rather than genetic predisposition. AML leads to immunosuppression and disruption of normal hematopoiesis, resulting in increased susceptibility to severe infections such as gram-negative septicemia. The systemic inflammatory response to infection damages the endothelium and activates the coagulation cascade, leading to disseminated intravascular coagulation (DIC). This consumptive coagulopathy causes depletion of natural anticoagulants, including protein C, resulting in acquired protein C deficiency. The deficiency impairs regulation of thrombin generation, allowing microvascular thrombosis and subsequent skin ischemia characteristic of purpura fulminans.

While congenital protein C deficiency due to genetic mutations can also cause purpura fulminans, it is unlikely in this adult patient without prior history, and the acute clinical setting supports an acquired etiology. The resulting septic shock-induced DIC was evidenced by the laboratory findings of severe thrombocytopenia (platelets as low as 6,000/μL), prolonged coagulation times, markedly elevated D-dimer (66,343 ng/mL) and thrombin-antithrombin complexes, and importantly, a reduced protein C activity (37%). These abnormalities underline the consumptive coagulopathy and the loss of natural anticoagulant pathways, particularly protein C, which is central to PF pathophysiology by permitting microvascular thrombosis and skin ischemia [1]. The coagulopathy was compounded by vasopressor-induced ischemia, which together precipitated acral necrosis and progressive gangrene of all four extremities-predominantly the lower limbs. The development of PF in this immunocompromised setting is a rare and severe complication, with limited cases reported in patients with relapsed AML complicated by gram-negative septicemia [12,13].

Furthermore, management was also complicated by conflicting hemostatic challenges. While anticoagulation is frequently employed to mitigate ongoing microvascular thrombosis in PF, the patient’s AML-associated chronic thrombocytopenia and antineoplastic therapy necessitated frequent platelet transfusions and forced intermittent withholding of anticoagulant therapy, illustrating the delicate balance required in this case. Her anemia and significant multi-organ dysfunction, including acute kidney injury and severe liver injury as reflected in elevated transaminases and bilirubin, further illustrate the systemic severity of her illness.

Surgical intervention was required for definitive management of ischemic tissue. The patient underwent a left below-the-knee amputation, with intraoperative findings of extensive dry gangrene throughout the foot progressing to wet gangrene near the ankle, edematous tissue, and small-caliber vessels with evidence of bleeding. Prior below-the-knee amputation later required revision with scapular-parascapular free flap coverage. These procedures provided wound control and tissue coverage while reducing the risk of secondary infection.

Further complicating the clinical course were superimposed opportunistic infections with multidrug-resistant organisms, including vancomycin-resistant *Enterococcus faecium *and* Scopulariopsis brevicaulis*, underscoring the vulnerability of patients with hematologic malignancies and the complexity of antimicrobial management in this population. A limitation of this case is the absence of protein C antigen concentration, FDP, and fibrinogen level measurements, which may have provided a more comprehensive characterization of the coagulopathy. However, the diagnosis of PF was supported by laboratory evidence of markedly reduced protein C activity, severely elevated D-dimer, thrombocytopenia, and abnormalities consistent with DIC. Future reports would benefit from incorporating these parameters to further elucidate the underlying coagulopathic mechanisms.

This case highlights the importance of maintaining a high index of suspicion for purpura fulminans in adult patients with sepsis and new skin necrosis, especially those with underlying malignancy. It also underscores the need for a multidisciplinary approach to address the complex diagnostic and therapeutic challenges posed by PF in immunocompromised patients. Overall, this patient’s clinical trajectory from relapsed AML and gram-negative septic shock to DIC and PF represents a rare but devastating complication that underscores the intersection of malignancy, infection, and coagulopathy, reflecting both the extreme fragility and complex care required in this population.

## Conclusions

PF is a severe complication characterized by rapidly progressing skin necrosis and DIC requiring early recognition by dermatologists and prompt multidisciplinary coordination for optimal patient outcomes. This case highlights its occurrence in an adult with relapsed AML and *E. coli *septicemia, where coagulopathy and immunosuppression complicated management, emphasizing the importance of timely resuscitation, targeted antimicrobial therapy, correction of coagulopathy, and surgical intervention. Practical clinical vigilance for new-onset purpura in septic and immunocompromised patients supports early diagnosis and management, which are important for improving prognosis. Multidisciplinary collaboration is essential in tailoring these interventions to individual patient needs.
